# Supplementary material for: NOD2 Agonism Counter-Regulates Human Type 2 T Cell Functions in Peripheral Blood Mononuclear Cell Cultures: Implications for Atopic Dermatitis
Source: Biomolecules. 2023 Feb 15;13(2):369. doi: 10.3390/biom13020369 (PMC9953199; doi:10.3390/biom13020369)
Supplement: Supplementary file 1 [file biomolecules-13-00369-s001.zip › biomolecules-2039614-supplementary.pdf]

biomolecules

Supplementary Materials

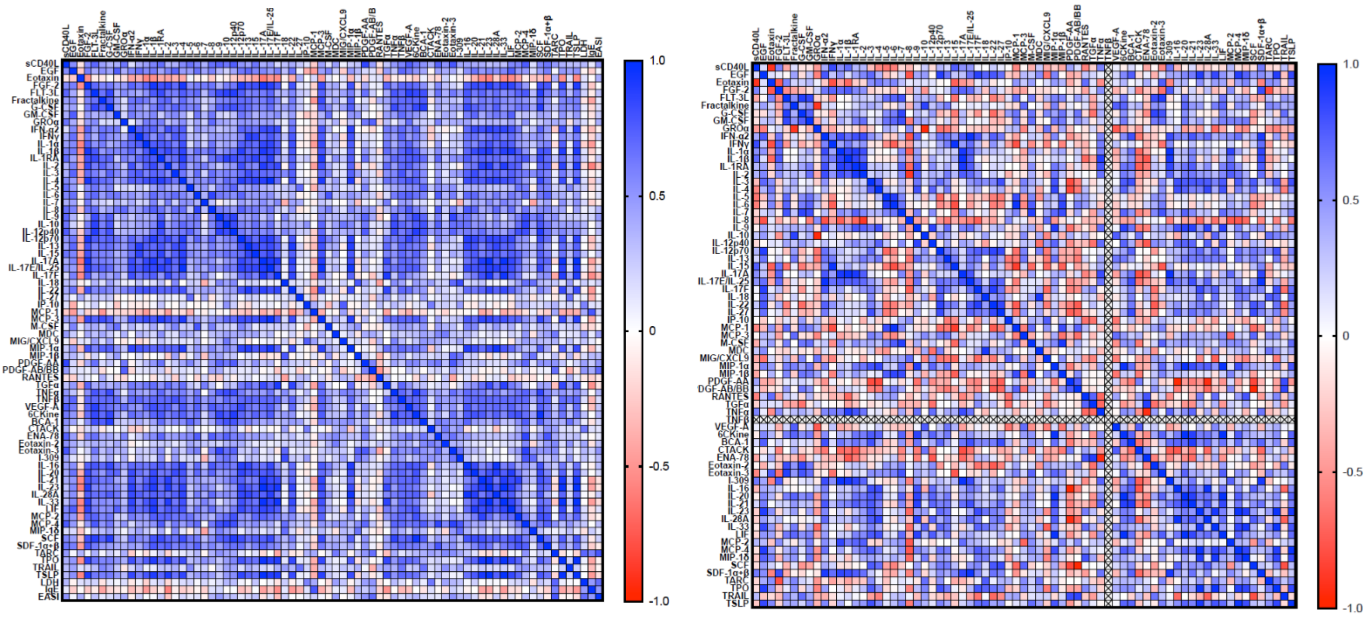

Figure S1. Correlation matrices of cytokines and chemokine profiles in blood, comparing blood from atopic dermatitis patients (**A**) versus healthy subjects (**B**). Spearman correlation matrix of median plasma cytokine and chemokine concentrations (pg/mL) for all 71 targets tested by immunoassay as described in Figure 1; moderate-to-severe adult AD patients (n = 15), matched healthy subjects (n = 7).

Table S1. Clinical parameters for atopic dermatitis patients and healthy subjects. Clinical and clinical laboratory variables are summarized for AD patients. Core clinician reported outcome measures, Eczema Area Severity Index (EASI, 4 signs [erythema, excoriation, swelling, lichenification] on 4 body sites, 0 = clear; 0.1 to 1.0 = almost clear; 1.1 to 7.0 = mild; 7.1 to 21.0 = moderate; 21.1 to 50.0 = severe; 50.1 to 72.0 = very severe disease), validated Investigator Global Assessment (vIGA™,range: 0=clear, 1=almost clear, 2=mild, 3=moderate, 4=severe, range: 0–100%), and body surface area (BSA) are indicated, in addition to routine serological biomarkers (total IgE and LDH; reference ranges: 0 to 200 kU/L, and 140 to 280 U/L respectively).

	**Atopic Dermatitis (n = 15)**		**Healthy subjects (n = 7)**	
**Clinical variables**	**median or sum**	**% or range**	**median or sum**	**%or range**
Age Age (yrs)	33	22–61	27	21–45
Sex (F)	10	66.7	4	57.1
Ethnicity (White)	9	60.0	3	42.9
Ethnicity (Asian)	5	33.3		
Ethnicity(Black)	1	6.7		
Ethnicity (Middle Eastern)	0	0.0		
Ethnicity (mixed, white-asian)	1	6.7		
*Lactate dehydrogenase (LDH, U/L) (n = 14)	221.5	151–482		
*IgE (ku/L)	2203.7	94.5-62235.8		
Eczema Area Severity Index (EASI)	23.6	11.6–54.0		
validated Investigator GI oba I Assessment (vIGA)	3	2.0–4.0		
Body surface area (BSA)	44	10–90		
Childhood onset	12	80.0		
Asthma	5	33.3		
Allergic rhinitis	9	60.0		
*Dust allergies (n = 14)	11	78.6		
*Animal allergies (n = 14)	8	57.1		
Food intolerance	6	42.9		
*FamilyHx AD (n = 14)	8	57.1		
*Family fix Asthma (n = 14)	3	21.4		
*Family Hx Allergic rhinitis (n = 14)	11	78.6		
